# Safety and Efficacy of Photon Re-Irradiation with Whole-Brain Radiotherapy or Craniospinal Irradiation for Recurrent Pediatric Central Nervous System Malignancies

**DOI:** 10.3390/curroncol33100617

**Published:** 2026-10-14

**Authors:** Palak P. Patel, Hesham Elhalawani, Karen J. Marcus, Lissa C. Baird, Katie P. Fehnel, Tom Rosenberg, Ralph Salloum, Ana Aguilar-Bonilla, Susan N. Chi, Karen D. Wright, Kee Kiat Yeo, Daphne A. Haas-Kogan, Kevin X. Liu

**Affiliations:** 1 Department of Radiation Oncology, Mass General Brigham Cancer Institute, Harvard Medical School, 55 Fruit St., Boston, MA 02114, USAkmarcus@mgb.org (K.J.M.);; 2 Department of Radiation Oncology, Dana-Farber Cancer Institute, Boston Children’s Hospital, Boston, MA 02115, USA; 3 Department of Neurosurgery, Boston Children’s Hospital, Boston, MA 02115, USA; 4 Department of Pediatric Oncology, Dana-Farber/Boston Children’s Cancer and Blood Disorders Center, Boston, MA 02115, USAralph_salloum@dfci.harvard.edu (R.S.);

**Keywords:** medulloblastoma, high grade glioma, ependymoma, pineoblastoma, non-germinomatous germ cell tumor, diffuse midline glioma

## Abstract

Background: Limited data exist regarding the safety and efficacy of photon re-irradiation with whole-brain radiotherapy (WBRT) or craniospinal irradiation (CSI) in children with recurrent or second primary CNS tumors. Materials and methods: In this retrospective single-institution study, 23 patients < 21 years who underwent photon re-irradiation with WBRT (*n* = 9) or CSI (*n* = 14) for CNS tumors between 2013 and 2026 were included. Clinical and treatment characteristics were collected. Progression-free survival (PFS) and overall survival (OS) were estimated using the Kaplan–Meier method. Toxicities were graded using CTCAE v5.0. Results: In our cohort, the median age at diagnosis was 8.5 years (range: 0.2–19.7 years), and medulloblastoma was the most common histology (30%). Median time to the first relapse after initial irradiation was 12.0 months (range: 0.9–92.5 months). Median re-irradiation dose to the whole brain or craniospinal axis was 24.0 Gy (range: 20.0–36.0 Gy). For 15 patients who received involved field boosts, median total dose was 30.6 Gy (range: 20.0–54.0 Gy). With median follow-up after re-RT of 12.2 months (range: 1.0–106.8), 12-month PFS and OS were 24.2% and 67.0%, respectively. Four patients who received curative-intent re-CSI remain alive with median follow-up of 20.4 months (range: 5.8–106.8). The crude rate of grade >2 CNS toxicity was 8.7%. Of 10 patients with new neurologic symptoms before re-irradiation, 7 (70%) had symptomatic improvement after treatment. Conclusions: Photon re-irradiation with WBRT or CSI was feasible and achieved disease control in a subset of patients and meaningful symptom palliation with acceptable acute toxicity. Careful attention to cumulative dose constraints is essential, and longer follow-up is needed to assess durability and late effects.

## Simple Summary

When central nervous system tumors recur in children after earlier radiation treatment, treatment options are limited, and outcomes are poor. We reviewed the experience of 23 children and young adults at our institution who received such a repeat course of photon radiotherapy for recurrent tumors. About two-thirds of patients were alive one year after treatment, and most patients whose symptoms had worsened before treatment, such as pain, weakness, difficulty walking, or blurred vision, improved afterward. Four patients treated with the goal of long-term disease control remain alive without their cancer growing. Serious radiation injury to the brain was uncommon, although low blood counts during craniospinal irradiation were frequent. These findings suggest that a second course of photon radiotherapy to the whole brain or the entire neuroaxis is a reasonable option for carefully selected children, both to relieve symptoms and, for some, to provide lasting control of their cancer.

## 1. Introduction

Relapsed pediatric central nervous system (CNS) malignancies carry a dismal prognosis, with survival often measured in months in the absence of effective salvage therapy. Approximately 30% of children with medulloblastoma experience relapse, which has historically been almost universally fatal, with median survival often less than 12 months despite salvage treatment [1]. Similar poor outcomes are observed in recurrent high-grade gliomas and other high-grade CNS tumors despite aggressive multimodality therapy [2,3]. In general, salvage options include repeat resection, systemic therapy, and in select cases, re-irradiation. Radiation oncologists have traditionally been cautious about delivering a second course of radiation due to concerns of severe toxicity such as radionecrosis or permanent neurocognitive injury, given that critical CNS structures may have received near-tolerance doses in initial therapy [4,5,6]. However, advances in radiotherapy techniques and growing clinical evidence have begun to challenge the notion that re-irradiation is prohibitive. Emerging evidence suggests that the brain and spinal cord have some capacity for repair between radiation courses, which can make carefully planned re-irradiation a viable option for select patients.

An increasing body of literature demonstrates potential benefit from re-irradiation in pediatric CNS malignancy recurrence. In recurrent medulloblastoma, small retrospective series report 1-year overall survival rates of 45–70% following re-irradiation, with outcomes particularly favorable in those with localized relapse, minimal residual disease, and longer intervals from initial treatment [1,7]. Long-term survivors have been reported after both focal re-irradiation and repeat craniospinal irradiation (CSI) [1], and multi-institutional data suggest a significant survival advantage when re-irradiation is incorporated into salvage therapy [7,8,9,10]. Re-irradiation has also shown promise in other pediatric CNS malignancies, including posterior fossa ependymoma, where re-irradiation with CSI has been associated with markedly improved disease control [3,6,11], and diffuse intrinsic pontine glioma, in which re-irradiation at progression can provide symptom relief and modest survival benefit with acceptable tolerability [2,12]. Collectively, these data support the feasibility of re-irradiation, even to large volumes such as the whole brain or craniospinal axis, using modern techniques. Beyond survival, symptom control is an important goal of re-irradiation in this population, as progressive disease can cause pain and declining neurologic function that may impair quality of life, and re-irradiation may provide meaningful palliation of these symptoms [2,12].

Despite these encouraging results, data specifically focused on large-field re-irradiation, such as whole-brain radiation therapy (WBRT) or CSI, remain limited in the pediatric population. Most published series have small sample sizes and often include only focal re-irradiation or stereotactic approaches for localized recurrences [1,2,3,7,9,10,13]. The largest series of re-irradiation with CSI was studied in patients with ependymoma, but all of these patients received focal radiotherapy at diagnosis [3]. Many of these studies also did not report dosimetric constraints. Tolerance of the developing CNS to a second course of high-volume radiation is not well-defined, and there remains concern regarding cumulative dose to organs at risk. Our study aims to characterize the clinical outcomes, including toxicity and symptom palliation, of photon re-WBRT or re-CSI in a cohort of pediatric patients with recurrent CNS malignancies and to correlate these outcomes with dosimetric parameters.

## 2. Methods

Institutional review board approval was obtained for this retrospective review of pediatric patients who underwent a second course of radiation with photon WBRT or CSI for recurrent CNS malignancies at Brigham and Women’s Hospital. Eligible patients were diagnosed with a primary CNS malignancy before age 21, received definitive radiotherapy (either focal brain or spine RT or CSI) as part of initial treatment, and subsequently received photon WBRT or CSI at the time of relapse or for a second malignancy in the CNS between January 2013 and August 2026. We excluded patients with less than one month of follow-up after the start of re-irradiation to ensure adequate time for toxicity assessment.

Data included demographics, tumor characteristics, initial therapy details, recurrence details, and re-irradiation parameters (field selection, dose, fractionation, and use of boosts) and were collected from the electronic medical record. Dosimetric data from initial and re-irradiation plans were used to calculate cumulative doses to selected organs at risk, including the whole brain, brainstem, optic chiasm, optic nerves, and spinal cord. Additional courses of radiotherapy delivered between the initial course and re-WBRT/re-CSI were recorded. The pattern of disease at the time of re-irradiation was classified based on imaging reports as diffuse leptomeningeal dissemination, multifocal nodular or parenchymal disease without diffuse leptomeningeal involvement, or localized recurrence with limited or no distant spread. Neurologic symptoms before and after re-irradiation, documented changes in cognition or mental status, systemic therapies (including molecularly targeted agents and immunotherapy), and available molecular and germline findings were abstracted from clinical documentation.

Re-irradiation was planned using computed tomography-based photon planning (Aria, Varian Medical Systems, Pao Alto, CA, USA) and was individualized by the treating radiation oncologist, accounting for the dose distribution of the prior radiotherapy course(s), interval since prior irradiation, extent of disease, and goals of care. Given the high cumulative doses anticipated from prior treatment, planning prioritized avoidance of the optic chiasm, optic nerves, and brainstem; when necessary, target coverage adjacent to these structures was intentionally reduced to limit their cumulative dose, accepting under-coverage of the prescription dose in these regions. In selected patients, the junction with a prior radiation field was also intentionally under-dosed to avoid overlapping high-dose regions.

STATA v15.0 (Stata Corp, College Station, TX, USA) and R (Version 4.6.1, R Core Team) were used for statistical analyses [14]. The primary endpoints were overall survival (OS) and progression-free survival (PFS), defined from the start of re-WBRT or re-CSI to death or the first subsequent progression, respectively. OS and PFS were analyzed using the Kaplan–Meier method. Baseline clinical and treatment characteristics were compared between the re-WBRT and re-CSI cohorts using Fisher’s exact test (with the Freeman–Halton extension for categorical variables with more than two levels) and the Mann–Whitney U test for continuous variables. Patients without an event were censored at the last follow-up. Secondary endpoints included patterns of failure and treatment-related toxicity. Recurrences were assessed relative to re-irradiation fields, with particular attention to failures in intentionally underdosed regions due to prior high-dose exposure. Toxicities were graded using CTCAE v5.0. Exploratory comparisons of PFS and OS according to histology (high-grade glioma or diffuse midline glioma [HGG/DMG] versus other histologies, and embryonal or germ cell tumors versus HGG/DMG versus ependymoma), disease pattern at re-irradiation (diffuse leptomeningeal dissemination versus localized or multifocal nodular disease), and receipt of systemic therapy before or after re-irradiation were performed using the log-rank test. Given the small sample size, multivariable analyses were not performed. A two-sided *p*-value < 0.05 was considered statistically significant.

## 3. Results

Twenty-three pediatric patients were identified, with a median age at initial diagnosis of 8.5 years (range: 0.2–19.7 years). Clinical characteristics are presented in Table 1. Baseline characteristics were largely similar between the re-WBRT and re-CSI cohorts. The most common histology was medulloblastoma (30%), followed by high-grade glioma (26%). Two patients had genetic predisposition, including Gorlin syndrome and Li-Fraumeni syndrome. At initial presentation, 14 patients (61%) received CSI as part of frontline therapy, whereas 9 patients (39%) received focal radiotherapy. The median initial radiation dose was 54 Gy (range: 21.6–59.4 Gy) to the tumor bed, and the median radiation dose to the craniospinal axis was 23.4 (range: 10.8–36.0 Gy). Most patients (70%) received proton radiotherapy for their initial course. Initial radiotherapy fields and doses for each patient, and any additional radiotherapy delivered between the initial course and re-irradiation, are listed in Supplementary Table S1.

All patients had radiographic evidence of progressive disease before undergoing re-irradiation, with a median interval of 12.0 months (range: 0.9–92.5 months) from completion of initial RT to first relapse. At first relapse, nine patients (39%) had recurrent disease confined to the brain (primary site and/or intracranial metastases), one patient (4%) had a second primary CNS embryonal tumor confined to the brain, four (17%) had disease only in the spine (spinal leptomeningeal dissemination without intracranial tumor), and nine (39%) had both intracranial and spinal disease. Salvage therapies before re-irradiation included surgery (*n* = 6, 26%), chemotherapy (*n* = 10, 43%), and additional focal radiation (*n* = 14, 61%) before ultimately receiving re-irradiation with WBRT/CSI (re-WBRT/re-CSI). Of the patients in the cohort, 7 patients (30%) received molecularly targeted agents (including MEK and BRAF, mTOR, smoothened, EZH2, tyrosine kinase, and KRAS G12C inhibitors and ONC201/dordaviprone) and 10 (43%) received immune-based regimens (immune checkpoint inhibitors, indoximod, or chimeric antigen receptor T-cell therapy) at some point during their treatment course.

All patients had evidence of disseminated disease on imaging at the time of re-CSI/re-WBRT. This included diffuse leptomeningeal dissemination in 17 patients (74%), multifocal nodular or parenchymal disease without diffuse leptomeningeal involvement in four (17%), and limited (oligometastatic) dissemination in two (9%) (Table 1). Of the 23 patients, 14 (61%) received re-CSI, and 9 (39%) received re-WBRT. Four patients (17%) received curative-intent re-CSI, and all other patients received palliative-intent re-irradiation. Eight patients (35%) received palliative hypofractionated re-CSI/re-WBRT with >2 Gy per fraction. The median dose to the whole brain or craniospinal axis was 24.0 Gy (range: 20.0–36.0 Gy). For 15 patients (65%), a simultaneously integrated or sequential boost was administered to areas of gross disease. The total re-irradiation dose to the involved field had a median of 30.6 Gy (range: 20.0–54.0 Gy). Maximum doses to critical normal structures from the re-irradiation course and cumulative maximum doses across all radiotherapy courses are shown in Table 2.

**Figure 1:**
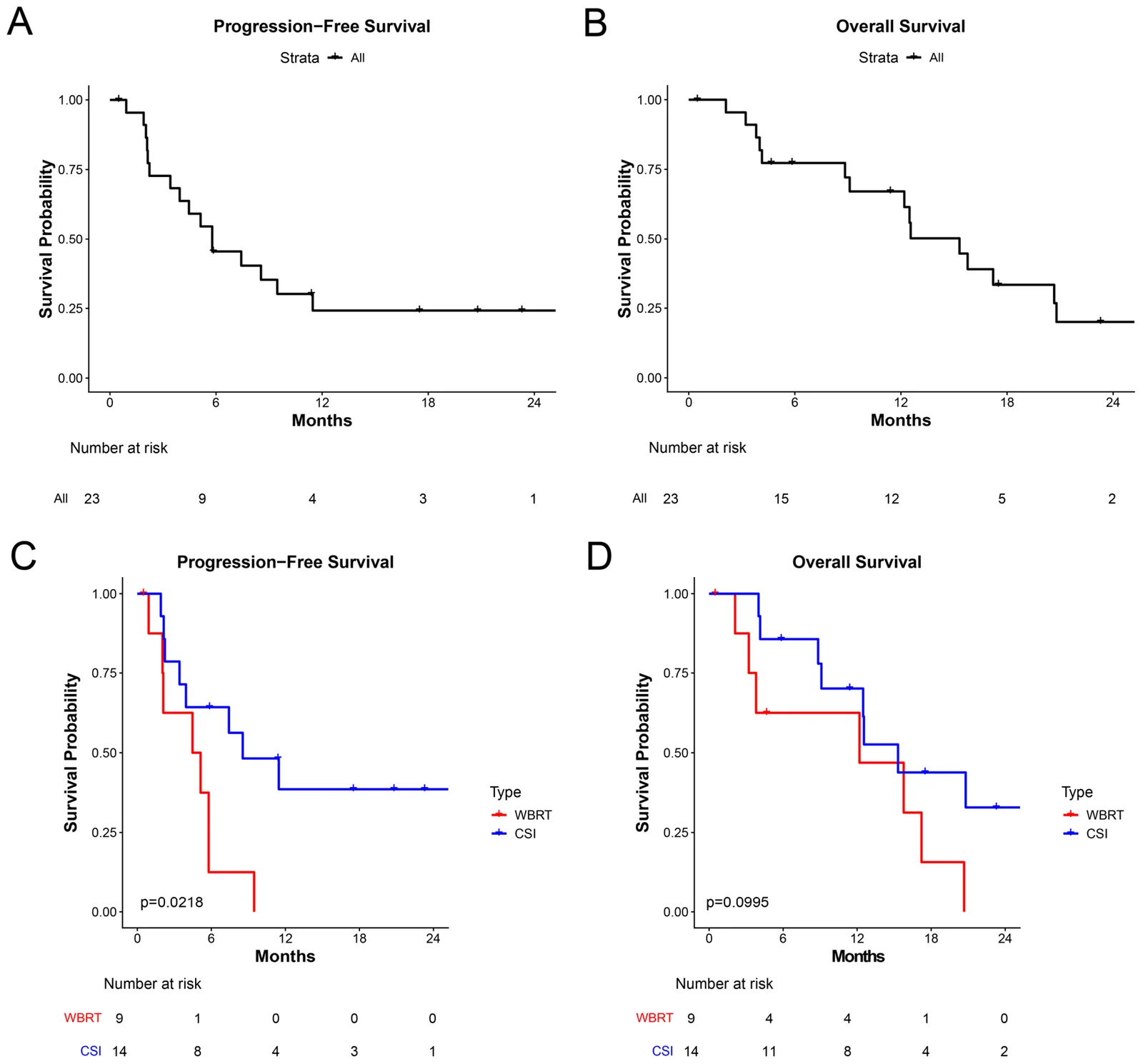
(**A**) Progression-free survival and (**B**) overall survival following re-irradiation with WBRT or CSI in the entire cohort; (**C**) progression-free survival and (**D**) overall survival stratified by re-WBRT versus re-CSI (log-rank *p*-values shown).

The median follow-up period after re-RT was 12.2 months (range: 1.0–106.8). Progression-free survival and overall survival at 12 months were 24.2% and 67.0%, respectively (Figure 1A). The median progression-free survival and overall survival were 5.8 months and 15.3 months, respectively (Figure 1B). While OS was not statistically different between the re-WBRT and re-CSI cohorts, PFS was improved in the re-CSI cohort (38.6% vs. 0.0% at 12 months, *p* = 0.02; Figure 1C,D). Notably, all four patients who received curative-intent re-CSI are alive without progression at median follow-up of 20.4 months (range: 5.8–106.8). Clinical and treatment details for these four patients are provided in Supplementary Table S2.

In exploratory analyses, patients with HGG/DMG (*n* = 6) had numerically inferior PFS compared with patients with other histologies (12-month PFS 0% vs. 31.8%; median PFS 3.9 vs. 7.4 months; *p* = 0.09), whereas OS did not differ significantly (12-month OS 53.3% vs. 70.1%; *p* = 0.24). When histologies were grouped as embryonal or germ cell tumors (*n* = 14), HGG/DMG (*n* = 6), and ependymoma (*n* = 3), PFS differed across groups but not significantly (*p* = 0.06), with no patient with ependymoma experiencing progression during available follow-up, and OS did not differ (*p* = 0.40). Patients with diffuse leptomeningeal dissemination at the time of re-irradiation (*n* = 17) had inferior PFS compared with those with multifocal nodular or oligometastatic disease (*n* = 6; 12-month PFS 14.7% vs. 60.0%; *p* = 0.04), and no significant difference in OS (12-month OS 56.5% vs. 100%; *p* = 0.08). Salvage systemic therapy before re-irradiation was not associated with PFS (*p* = 0.43) or OS (*p* = 0.75). Among 22 patients with available data, those who received systemic therapy after re-irradiation (*n* = 13) had longer OS than those who did not (*n* = 9; 12-month OS 84.6% vs. 37.5%; *p* = 0.007), without a significant difference in PFS (*p* = 0.07).

In terms of symptom control, of the 10 patients who developed new symptoms with disease progression before re-irradiation, seven had improvement of symptoms after re-CSI or re-WBRT, and three patients’ symptoms remained stable. Symptom improvement after re-CSI/re-WBRT spanned pain, urinary function, gait/motor strength, cranial nerve deficits, and mental status. A patient with LMD and a cauda equina mass had resolution of leg and sacral pain with subsequent Foley removal. Three patients showed functional gains and regained the ability to ambulate independently after presenting with lower extremity weakness. One patient with extensive LMD causing dysphagia, facial weakness, and gait instability stabilized post-RT with only minor fatigue. A patient presenting with depressed mental status regained the ability to follow commands and speak after dexamethasone and RT. Another patient receiving WBRT for LMD-related blurry vision had improved vision on treatment.

**Figure 2:**
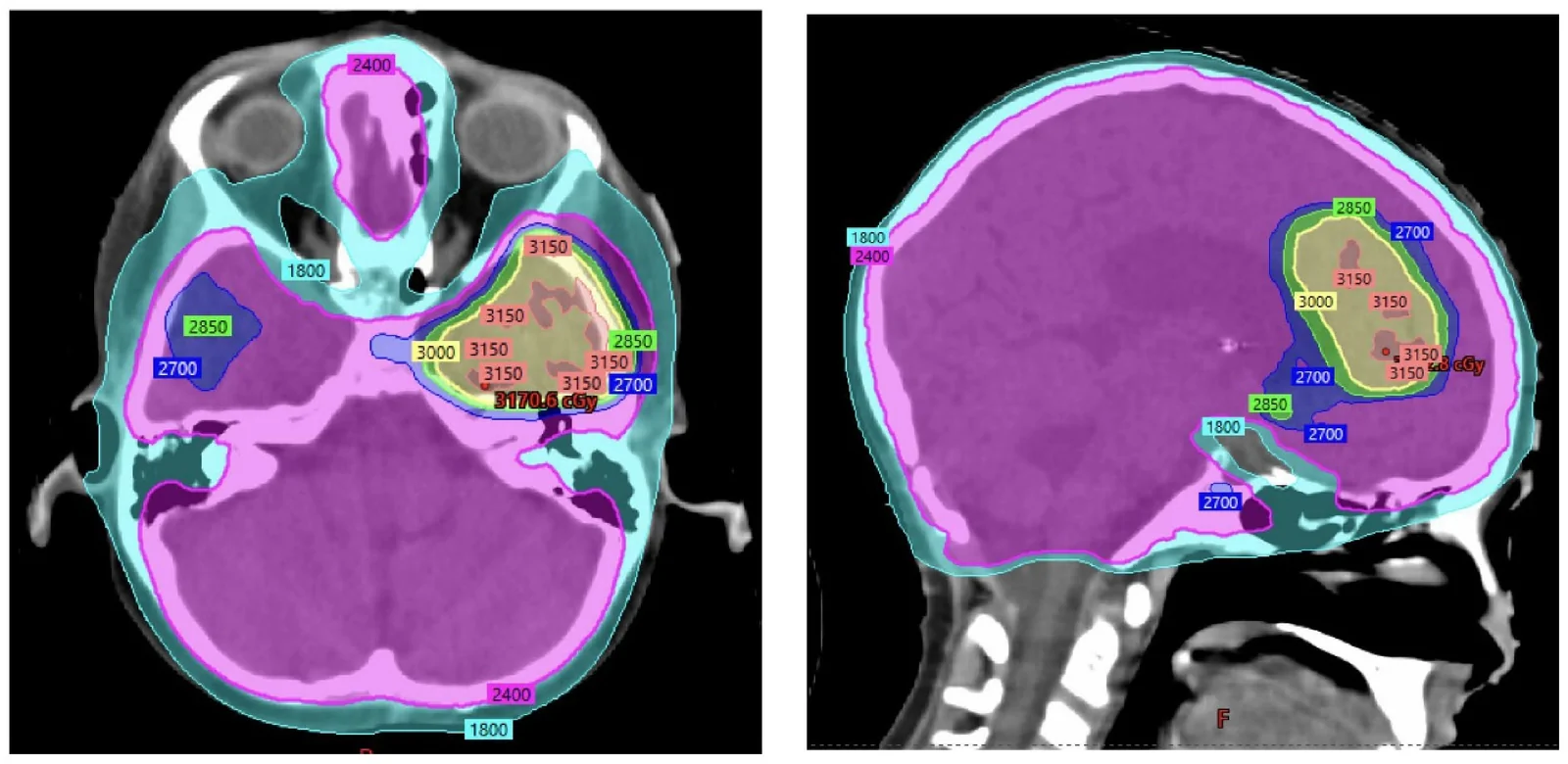
Representative re-irradiation treatment plan demonstrating undercoverage of prescription near the optic nerves and chiasm to prioritize sparing of organs at risk. Color wash indicates isodose levels in cGy.

For 15 patients who relapsed after re-WBRT/re-CSI, 9 (60%) relapsed within the prior RT field, 4 (27%) were outside the RT field, and 2 (13%) had both in- and out-of RT field recurrences. In seven patients, the treating radiation oncologist intentionally underdosed at the match with a prior radiation field. Among these patients, four later developed tumor recurrence at the field junction. Ten patients (43%) had underdosing around the optic chiasm and optic nerves, and none of these patients had recurrences within the underdosed region around the optic chiasm and optic nerves (Figure 2).

**Figure 3:**
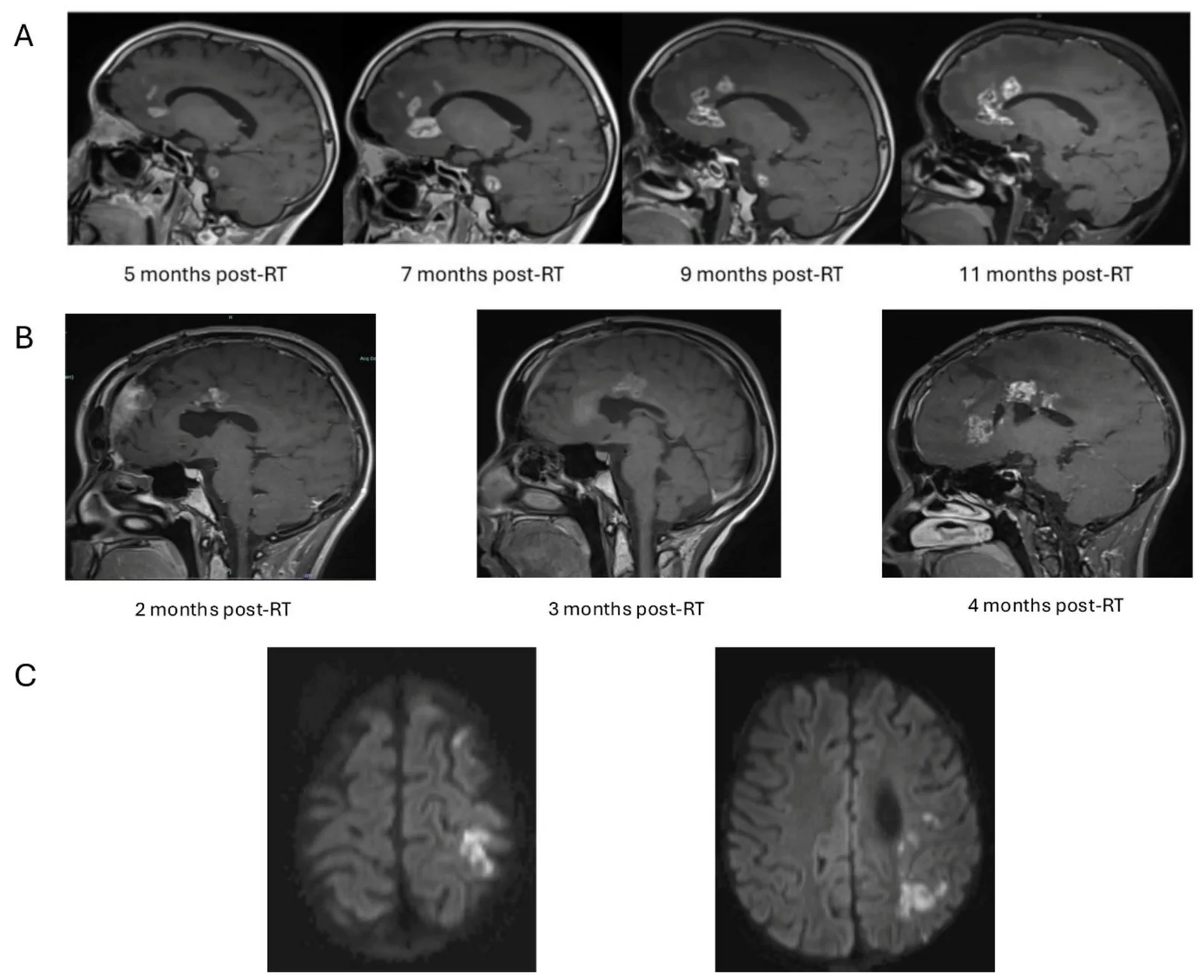
(**A**) Serial MRI demonstrating a patient with asymptomatic radionecrosis after re-irradiation with interval improvement without intervention. (**B**) Serial MRI demonstrating progression of a patient’s symptomatic radionecrosis after re-irradiation, and the patient required steroids and bevacizumab with good effect. (**C**) Axial diffusion-weighted MRI for a patient demonstrating multifocal areas of restricted diffusion consistent with evolving infarcts.

Re-irradiation was generally well tolerated. On imaging follow-up, none of the patients showed radiographic evidence of injury to the optic nerves, and no patients had vision loss after re-irradiation. Two patients had brain necrosis noted on imaging, with one patient who, after re-CSI for disseminated supratentorial ependymoma, had a cumulative maximum brain dose of 119.9 Gy, developed grade 3 brain necrosis with left upper extremity weakness; they received steroids and bevacizumab, with improvement. The other patient with a cumulative maximum brain dose of 89.6 Gy after re-CSI for disseminated non-germinomatous germ cell tumor developed asymptomatic grade 1 brain necrosis that improved over time without any intervention. One patient who received anti-angiogenic systemic therapy after re-CSI for disseminated posterior fossa ependymoma developed vasculopathy complicated by a stroke 16 months after re-irradiation, followed by development of secondary acute myeloid leukemia (AML) 19 months after re-irradiation, and ultimately passed due to complications at 20.8 months after re-irradiation without disease progression (Figure 3). Autopsy demonstrated atypical immature hematopoietic cells within rare vessels with associated vascular occlusion and hemorrhage, alongside hypoxic–ischemic injury, diffuse cerebral edema, and patchy subacute infarcts; it remains unclear whether the hematologic malignancy contributed to the patient’s demise. Another patient who had prior anti-angiogenic systemic therapy and multiple courses of focal brain and spine RT, and ultimately received re-WBRT, developed secondary AML 3 months after re-irradiation and ultimately died from progressive disease 12.5 months after re-WBRT while in remission from AML. The crude incidence of grade ≥ 2 CNS toxicity was 8.7%.

Clinically, some patients reported mild acute symptoms during the re-irradiation course, such as grade 1 headaches or fatigue, which were managed conservatively. Transient exacerbation of pre-existing neurologic deficits was noted in one patient after the first fraction, but this resolved to baseline status with steroids, and the steroids were eventually weaned. Among the 14 patients who underwent re-irradiation with CSI, 10 had regular hematologic monitoring during treatment and within two weeks of completing CSI, and all 10 evaluable patients had grade ≥3 acute hematologic toxicities (Table 3). Six of these patients received support with packed red blood cell transfusions and platelet transfusions. With regard to endocrine function, no patients developed new deficits in the time period after re-CSI/re-WBRT. Formal neuropsychological testing was not routinely performed after re-irradiation; however, on review of clinical documentation, no patient had serious neurocognitive or mental status deterioration attributed to re-irradiation, and one patient with progression-related depressed mental status improved after treatment. The longest surviving patient has neurocognitive late effects requiring educational supports, likely reflecting cumulative treatment effect. Nonetheless, neurocognitive outcomes are difficult to assess given limited follow-up for most patients.

## 4. Discussion

In this retrospective study, we evaluated pediatric patients who underwent re-irradiation with WBRT or CSI for recurrent CNS malignancies. Our findings demonstrate that this aggressive approach is feasible and can yield meaningful disease control and symptom relief in heavily pre-treated pediatric patients with minimal acute toxicity [15]. While most patients ultimately experienced further progression, a subset achieved more durable remissions, with one patient maintaining disease control for over 8 years. To our knowledge, this represents one of the largest reports focusing specifically on photon WBRT/CSI salvage re-irradiation for pediatric CNS malignancies [1,6,10].

Our cohort’s outcomes with 12-month PFS of 24.2% and OS of 67% after salvage CSI/WBRT re-irradiation are consistent with those reported in the literature [1,8,9,10,15]. Outcomes for relapsed medulloblastoma and other pediatric embryonal tumors have been extremely poor, with median survival often approximately 6 months without definitive salvage treatment [15]. With modern multimodal regimens, including resection, high-dose chemotherapy, and re-irradiation, improvement in outcomes has been reported. Bakst et al. reported a median OS of 30 months after re-RT in medulloblastoma patients, most of whom received focal re-RT and one patient who received re-CSI, with 5-year OS ~65% in their selective subset [1]. Wetmore et al. demonstrated that incorporating re-irradiation into relapse therapy significantly improved long-term survival for both standard- and high-risk medulloblastoma patients, with 10-year OS ~33% in re-irradiated patients versus 0% without further RT [10]. Song et al. recently published a series of 54 pediatric patients receiving proton re-irradiation for recurrent CNS tumors, including 20 patients who received re-CSI, and found that median PFS was 16.5 months and OS was 46 months. The study did not report the percentage of patients with disseminated recurrence before re-irradiation and included a high percentage of patients receiving curative-intent radiation (72%), which may contribute to the more favorable outcomes [16]. Nonetheless, these data underscore that re-irradiation can be a critical component of salvage, and there may be a subset of patients who can be long-term survivors. In contrast, patients undergoing re-irradiation for aggressive histologies, such as high-grade gliomas, or early recurrence following prior treatment experienced more limited benefit, supporting prior observations that re-irradiation may be most effective when delivered in settings where disease can be treated more definitively [1,17].

Within our cohort, most patients recurred within the RT field, which is in line with prior studies. A study regarding patterns of relapse after re-irradiation for medulloblastoma found that a significant portion of failures after re-RT were in-field (up to ~50%) [1], highlighting the challenge of achieving durable local control while balancing the need for meaningful tumor dose against cumulative toxicity, particularly when re-irradiation is delivered with palliative intent. Furthermore, Tsang et al. reported that treating the whole neuraxis at recurrence for ependymomas dramatically reduced subsequent relapse in untreated areas, suggesting that when there was propensity to disseminate, focal re-irradiation may be insufficient [3]. In our study, we found that PFS was significantly improved for patients receiving reCSI, suggesting that comprehensive neuroaxis re-irradiation may help delay or limit out-of-field recurrences. In addition, we observed that of the seven patients with radiation plans that incorporated a matched line to prior radiation fields, four patients had recurrences at the field junction. On the other hand, the optic nerves and chiasm were frequently underdosed, and no patients in our cohort had loss of vision after re-WBRT/re-CSI, and there were no recurrences along the optic nerves or chiasm. This highlights a central tension in re-irradiation planning: balancing normal tissue constraints with adequate tumor coverage.

Symptom palliation was an important benefit of re-irradiation in our cohort. Of the 10 patients who developed new neurologic symptoms with disease progression prior to re-irradiation, 7 experienced symptomatic improvement after re-CSI/re-WBRT, spanning pain, urinary function, gait and motor strength, cranial nerve deficits, and mental status. Notable examples included resolution of pain and urinary retention sufficient to allow Foley catheter removal, recovery of ambulation in patients with pre-treatment weakness and falls, and improved alertness and command-following in a patient with progression-related depressed mental status. These findings suggest that even when durable disease control is not achieved, re-irradiation can meaningfully improve neurologic function and quality of life in the short term, which is a clinically important outcome in a population with otherwise limited options.

Two patients (8.7%) developed grade ≥ 2 neurologic adverse events after re-irradiation, with one patient developing necrosis and another patient developing vasculopathy and stroke after re-CSI and anti-angiogenic therapy. Tsang et al. reported a 10-year cumulative incidence of grade ≥ 3 radiation necrosis of 7.9% after re-irradiation for ependymomas [6]. Similarly, in the large Pediatric Normal Tissue Effects in the Clinic (PENTEC) review of pediatric re-irradiation, the pooled incidence of imaging-confirmed necrosis was around 5% for brain and 3% for brainstem at a median of 2.3 years after re-irradiation [18]. Several other retrospective studies of pediatric patients undergoing CNS re-irradiation reported <2% of patients developing radiation necrosis, though these studies included focal re-irradiation, which may carry different risks from re-CSI/reWBRT [9]. Modern conformal techniques undoubtedly contributed to sparing of normal tissue. Additionally, the interval between radiation courses is known to impact tolerance. The median interval of approximately 1 year in our group between initial radiotherapy and relapse may have allowed partial recovery of normal tissue reserve. Our cohort’s median follow-up of 12.2 months is within the typical latency for radiation necrosis, though many patients progressed and died within 1 year of re-WBRT/re-CSI; thus, it is possible that some patients simply did not live long enough to manifest late effects. Thus, our toxicity results must be interpreted with caution and will likely evolve with longer follow-up in patients who are alive beyond 1 year. Of note, two patients who received anti-angiogenic therapy and spine RT developed secondary AML, a finding that has been reported in previous studies [11,19,20,21]. These data suggest that even in the setting of poor prognoses, secondary cancer risk should not be understated when counseling patients and families.

Neurocognitive and endocrine effects are of particular concern in pediatric re-irradiation. While no patient had documented serious cognitive or mental status deterioration attributed to re-irradiation during short-term follow-up, our longest surviving patient has neurocognitive and endocrine late effects after two courses of CSI. This is consistent with data from survivors of repeat CSI indicating that learning difficulties and hormonal deficiencies are common [3,8,22]. Almost all long-term pediatric brain tumor survivors experience some degree of neurocognitive slowing after radiation, and a second course could compound this effect. Tsang et al. found that nearly all children needed assistance in school and half had endocrinopathies after re-CSI [3]. In the Baroni et al. study of re-CSI for medulloblastoma, survivors fell within the mild to moderate intellectual disability range [8]. Thus, it is a limitation of our study that, given the small number of patients alive >2 years after re-WBRT/re-CSI, late effects could not be characterized.

While re-irradiation may be undertaken more readily in palliative settings where symptom control and quality of life are the primary goals, there may also be select children for whom re-WBRT or re-CSI offers a realistic opportunity for durable disease control or cure. In these cases, re-irradiation should be carefully considered and discussed with families, acknowledging the risk of serious late complications. Our findings align with the growing consensus that prior radiation is not a contraindication to further radiation in pediatrics, especially in the era of highly conformal planning and improved understanding of dose tolerances [13]. The PENTEC consortium and other collaborative efforts are beginning to establish guidelines on normal tissue dose-volume effects in children, including in the re-irradiation setting [18,22]. As these guidelines mature, they will help clinicians better predict which patients are good candidates for re-RT and how to plan treatments to maximize tumor control while avoiding toxicity.

Our findings also provide insight into which patients may benefit most from large-field re-irradiation. Embryonal tumors, such as medulloblastoma, and germ cell tumors are generally more radiosensitive than diffuse gliomas. In our exploratory analyses, patients with HGG/DMG had numerically inferior PFS compared with patients with other histologies, and none of the three patients with ependymoma progressed after re-CSI, although the small number of patients in each group limited statistical power. These observations are consistent with the series by Song et al., in which patients with glioma had inferior outcomes after proton re-irradiation compared with those with ependymoma [16], and with reports in DMG demonstrating that re-irradiation improves symptoms and modestly prolongs survival but rarely provides durable disease control [12,23,24]. In a recent series of 27 children with recurrent medulloblastoma, most of whom received CSI as part of re-irradiation, 2-year OS was 44% [25], and outcomes after re-irradiation for medulloblastoma appear most favorable in patients with localized relapse and lower disease burden [1,7]. Consistent with these data, patients in our cohort with diffuse leptomeningeal dissemination at the time of re-irradiation had inferior PFS compared with those with localized or multifocal nodular disease, although this association was likely confounded by treatment intent. Receipt of systemic therapy after re-irradiation was associated with improved OS, but this likely reflects selection of patients with better performance status and more favorable disease for further therapy. Taken together, these data suggest that curative-intent re-CSI may be most appropriate for patients with radiosensitive histologies and localized or lower-volume recurrence, and re-irradiation for HGG/DMG or diffuse leptomeningeal disease is primarily palliative.

The feasibility of large-field re-irradiation likely also reflects changes in frontline radiotherapy. CSI doses for average-risk medulloblastoma have been reduced from 36 Gy to 23.4 Gy with the addition of chemotherapy; 18 Gy has been evaluated in young children with inferior event-free survival in ACNS0331 [26]. Lower initial CSI doses may preserve normal tissue tolerance for a second course. In addition, 70% of our patients received proton therapy initially. Proton CSI avoids exit dose to the thoracic, abdominal, and pelvic organs and causes less hematologic toxicity than photon CSI [27], all of which may contribute to the feasibility of re-irradiation. A report from Children’s Hospital of Philadelphia reported encouraging survival and low rates of severe late toxicity among 54 children treated with proton re-irradiation, including re-CSI [16], and favorable results have been reported for tumors adjacent to the brainstem [28]. In a recent series of 41 children with recurrent ependymoma, most of whom had received proton therapy initially, proton re-irradiation achieved a 5-year OS of 64% with only one grade 3 toxicity [4,29]. In contrast, in a 30-year experience of 150 children re-irradiated for recurrent ependymoma, necrosis was more frequent after proton than photon re-irradiation (50.8% vs. 31.4%), and grade 3 necrosis was highest among patients treated with protons for both courses (31.7% vs. 12.3% with photons for both courses) [30]. Our data add to the growing re-irradiation literature and demonstrate that photon re-WBRT/re-CSI is a feasible option for select patients at relapse. Future studies are needed to further characterize the benefits and risks of proton and photon re-irradiation for relapsed or secondary pediatric CNS tumors.

There are several limitations to our study. This remains a small cohort of patients comprising multiple tumor types, several of which were represented by only one or a few patients, which limits statistical power and precludes definitive histology-specific conclusions. The heterogeneity of histologies, which differ in radiosensitivity, and prior treatments in our cohort is another limitation; outcomes likely differ substantially by tumor biology [6,7]. Additionally, as a retrospective analysis, there is inherent selection bias because patients who underwent re-irradiation were carefully chosen, likely those with better performance status and longer initial remission. This could overestimate the efficacy of re-irradiation compared to an unselected population of relapsed patients. In addition, comprehensive molecular characterization was not uniformly available, as many patients were diagnosed before routine DNA methylation profiling and next-generation sequencing; we were therefore unable to evaluate outcomes according to molecular subgroup or to identify biomarkers that could guide the integration of targeted therapy with re-irradiation. During their disease course, seven patients (30%) received molecularly targeted agents (including MEK and BRAF, mTOR, smoothened, EZH2, tyrosine kinase, and KRAS G12C inhibitors and ONC201/dordaviprone), ten (43%) received immunotherapy-based regimens (immune checkpoint inhibitors, indoximod, or chimeric antigen receptor T-cell therapy); however, the heterogeneous timing of these therapies relative to re-irradiation precluded assessment of their contribution to outcomes. As molecularly targeted therapies, such as ONC201 for H3 K27M-altered diffuse midline glioma, become more widely available [31], future studies should incorporate molecular classification and evaluate the integration of targeted agents with re-irradiation. The follow-up in many patients was relatively short due to the poor prognosis, limiting our ability to assess long-term outcomes and late toxicity. It is important to note that physician or family preference regarding radiation fields, including consideration of curative-intent and palliative-intent treatment, cannot be fully captured within our retrospective study; thus, there is likely selection bias regarding patients who received re-CSI, likely contributing to the improved outcomes in this cohort. Prospective data on pediatric re-irradiation are needed, though challenging to obtain given the rarity and urgency of the situation at relapse. Incorporating advanced technologies might further expand the therapeutic window by reducing normal tissue re-exposure [17]. Finally, quality of life assessments should be included in future studies to better characterize the functional and patient-centered impact of re-irradiation.

## 5. Conclusions

Photon re-irradiation with WBRT or CSI is a feasible salvage strategy for children and young adults with recurrent CNS malignancies, providing symptom palliation in most symptomatic patients and disease control in a subset, with acceptable toxicity. Four patients treated with curative intent remain alive without progression, suggesting that carefully selected patients, particularly those with radiosensitive histologies and localized or lower-volume recurrence, may achieve durable disease control. These results add to the accumulating evidence that even extensive re-irradiation can be undertaken safely in children, offering some patients symptomatic improvement and, in select cases, prolonged survival or potential cure [3,10]. Outcomes were less favorable for patients with HGG/DMG and those with diffuse leptomeningeal dissemination, for whom re-irradiation is primarily palliative. Careful attention to cumulative dose to critical structures is essential, and hematologic toxicity is common after re-CSI. Given the poor prognosis of recurrent pediatric brain tumors, aggressive approaches like re-irradiation warrant consideration within a multidisciplinary framework. Ongoing follow-up of our cohort will further elucidate the durability of disease control and late effects, and prospective studies remain needed to better define the role of re-irradiation in the management of these challenging recurrences.

## Figures and Tables

**Table 1:** Patient clinical and treatment characteristics.

Characteristic	All Patients N (%) or Median (Range) (*n* = 23)	reWBRT N (%) or Median (Range) (*n* = 9)	reCSI N (%) or Median (Range) (*n* = 14)	*p*-Value
Sex				1.000
Female	14 (61%)	5 (56%)	9 (64%)	
Male	9 (39%)	4 (44%)	5 (36%)	
Age at diagnosis	8.5 years (Range: 0.2–19.7)	8.7 years (Range: 4.9–11.8)	6.3 years (Range: 0.2–19.7)	0.431
Genetic predisposition syndrome				1.000
No	21 (91%)	8 (89%)	13 (93%)	
Yes	2 (9%)	1 (11%)	1 (7%)	
Histology at diagnosis				0.500
Medulloblastoma	7 (30%)	4 (44%)	3 (21%)	
High-grade glioma/diffuse midline glioma	6 (26%)	3 (33%)	3 (21%)	
Pineoblastoma	4 (17%)	1 (11%)	3 (21%)	
Ependymoma	3 (13%)	0 (0%)	3 (21%)	
ATRT	1 (4%)	0 (0%)	1 (7%)	
CNS embryonal tumor, NOS	1 (4%)	1 (11%)	0 (0%)	
Pure germinoma	1 (4%)	0 (0%)	1 (7%)	
Extent of Resection at initial diagnosis				0.081
GTR	10 (43%)	1 (11%)	9 (64%)	
NTR	4 (17%)	2 (22%)	2 (14%)	
STR	3 (13%)	2 (22%)	1 (7%)	
Biopsy	5 (22%)	3 (33%)	2 (14%)	
No surgery	1 (4%)	1 (11%)	0 (0%)	
Systemic therapy before RT				1.000
No	18 (78%)	7 (78%)	11 (79%)	
Yes	5 (22%)	2 (22%)	3 (21%)	
Type of first RT				0.228
CSI	14 (61%)	7 (78%)	7 (50%)	
Focal	9 (39%)	2 (22%)	7 (50%)	
Concurrent systemic therapy with RT				0.179
No	15 (65%)	4 (44%)	11 (79%)	
Yes	8 (35%)	5 (56%)	3 (21%)	
Systemic therapy after RT				0.228
No	9 (39%)	2 (22%)	7 (50%)	
Yes	14 (61%)	7 (78%)	7 (50%)	
Initial RT modality				0.363
Proton	16 (70%)	5 (56%)	11 (79%)	
Photon	7 (30%)	4 (44%)	3 (21%)	
First RT cumulative total dose	54 Gy/GyRBE (range: 21.6–59.4)	54 Gy/GyRBE (Range: 36.0–59.4)	54 Gy/GyRBE (Range: 21.6–59.4)	0.747
Site of first recurrence/second primary CNS tumor				0.741
Brain only	10 (43%)	3 (33%)	7 (50%)	
Spine only	4 (17%)	2 (22%)	2 (14%)	
Brain and spine	9 (39%)	4 (44%)	5 (36%)	
Disease pattern at reWBRT/reCSI				1.000
Leptomeningeal dissemination	17 (74%)	7 (78%)	10 (71%)	
Multifocal nodular disease	4 (17%)	1 (11%)	3 (21%)	
Oligometastatic disease	2 (9%)	1 (11%)	1 (7%)	
Salvage therapy before reWBRT/reCSI				1.000
Surgery	6 (26%)	2 (22%)	4 (29%)	
Chemotherapy	10 (43%)	4 (44%)	6 (43%)	
Focal RT	14 (61%)	5 (56%)	9 (64%)	

**Table 2:** Re-irradiation course and cumulative dosimetric parameters for organs at risk.

Organ at Risk	Re-Irradiation Dmax (Gy), Median (Range)	Cumulative Dmax (Gy), Median (Range)	Cumulative Dmean (Gy), Median (Range)
Brain	35.6 (21.4–58.2)	94.3 (60.1–124.9)	59.8 (27.6–71.0)
Brainstem	25.6 (13.4–56.9)	80.7 (42.7–113.3)	73.7 (37.3–100.3)
Optic chiasm	20.6 (7.1–46.8)	57.5 (43.8–104.2)	55.5 (22.7–72.5)
Left optic nerve	24.0 (8.0–44.3)	55.0 (30.2–103.6)	48.7 (27.7–63.3)
Right optic nerve	24.4 (8.0–45.6)	55.1 (30.0–105.2)	47.0 (27.1–63.2)
Spinal cord	24.7 (5.0–51.6)	58.1 (25.0–112.6)	37.2 (0.5–63.6)

**Table 3:** Acute hematologic toxicities during CSI re-irradiation (*n* = 10).

Toxicity	Grade 0	Grade 1	Grade 2	Grade 3	Grade 4
Leukopenia	0 (0%)	0 (0%)	5 (50%)	5 (50%)	0 (0%)
Neutropenia	1 (10%)	3 (30%)	3 (30%)	3 (30%)	0 (0%)
Lymphopenia	0 (0%)	0 (0%)	0 (0%)	6 (60%)	4 (40%)
Anemia	4 (40%)	0 (0%)	4 (40%)	2 (20%)	0 (0%)
Thrombocytopenia	1 (10%)	3 (30%)	2 (20%)	3 (30%)	1 (10%)

## Data Availability

Data are available upon reasonable request due to privacy/ethical restrictions.

## Supplementary Materials

The following supporting information can be downloaded at: https://www.mdpi.com/article/10.3390/curroncol33100617/s1, Table S1: Initial radiotherapy doses to the brain and spine, additional radiotherapy delivered between the initial course and re-irradiation, and re-irradiation for all 23 patients; Table S2: Characteristics, treatment details, and outcomes of the four patients treated with curative-intent re-CSI.

## Abbreviation

AML	Acute myeloid leukemia
CNS	Central nervous system
CSI	Craniospinal irradiation
CTCAE	Common Terminology Criteria for Adverse Events
DMG	Diffuse midline glioma
HGG	High-grade glioma
LMD	Leptomeningeal disease
PENTEC	Pediatric Normal Tissue Effects in the Clinic
Re-WBRT	Reirradiation with whole-brain radiotherapy
Re-CSI	Reirradiation with craniospinal irradiation
WBRT	Whole-brain radiotherapy
