# Supplementary material for: Safety and Efficacy of Photon Re-Irradiation with Whole-Brain Radiotherapy or Craniospinal Irradiation for Recurrent Pediatric Central Nervous System Malignancies
Source: Curr Oncol. 2026 Oct 14;33(10):617. doi: 10.3390/curroncol33100617 (PMC13703033; doi:10.3390/curroncol33100617)
Supplement: Supplementary file 1 [file curroncol-33-00617-s001.zip › curroncol-4564855-supplementary.pdf]

Supplementary Materials

Safety and Efficacy of Photon Re-Irradiation with Whole Brain Radiotherapy or Craniospinal Irradiation for Recurrent Pediatric Central Nervous System Malignancies

Table S1. Initial radiotherapy doses to the brain and spine, additional radiotherapy delivered between the initial course and re-irradiation, and re-irradiation for all 23 patients.

Pt	Histology	Initial RT			Additional RT between initial RT and re-irradiation		Re-irradiation (dose/dose per fraction)
		Technique	Brain	Spine	Brain	Spine	
**Re-CSI (n = 14)**							
1	Medulloblastoma, D/N	Proton CSI	18 GyRBE; PF 23.4 GyRBE; boost to 54 GyRBE	18 GyRBE	None		CSI 30.6 Gy/1.8 Gy; boost to 54 Gy
2	Medulloblastoma	Proton CSI	23.4 GyRBE; boost to 54 GyRBE	23.4 GyRBE	None		CSI 23.4 Gy/1.8 Gy; boost to 41.4 Gy
3	Medulloblastoma	Proton CSI	23.4 GyRBE; boost to 54 GyRBE	23.4 GyRBE	—	7.2 Gy/3 fx (T4–L5; urgent, 6 days before re-CSI)	CSI 24 Gy/2 Gy; SIB to 36 Gy/3 Gy
4	Spinal HGG (MN1-altered)	Proton focal	—	54 GyRBE (T12–L3)	Proton WBRT 36 GyRBE; boost to 54 GyRBE (right cerebellum)	Proton 21.6-36 GyRBE (C1-T11) boost to 50.4 Gy boost (T10); photon 35.25 Gy/15 fx (T12–sacrum)	CSI 24 Gy/2 Gy; SIB to 30 Gy/2.5 Gy
5	HGG (H3/IDH wild-type)	Photon focal	59.4 Gy (right temporal lobe)	—	None		CSI 30 Gy (brain)/36 Gy (spine), 3 Gy/fx
6	Spinal DMG, H3 K27-altered	Photon focal	—	54 Gy (C3–T7)	—	39 Gy/13 fx (C1); 39 Gy/13 fx (Brainstem); 4 Gy/1 fx (T1–L2)	CSI 20 Gy/4 Gy
7	Pineoblastoma	Proton CSI	23.4 GyRBE; boost to 54 GyRBE	23.4 GyRBE	None		CSI 30.6 Gy/1.8 Gy; SIB to 45 Gy
8	Pineoblastoma	Proton CSI	23.4 GyRBE; boost to 54 GyRBE	23.4 GyRBE	None		CSI 30.6 Gy/1.8 Gy; boost to 54 Gy/2.16 Gy (45 Gy at brainstem)
9	Pineoblastoma	Proton CSI	23.4 GyRBE; boost to 54 GyRBE	23.4 GyRBE	39 Gy/13 fx (left temporal lobe)	—	CSI 23.4 Gy/1.8 Gy
10	PF ependymoma	Proton focal	54 GyRBE (posterior fossa)	—	None		CSI 30 Gy/3 Gy; SIB to 35 Gy/3.5 Gy
11	Supratentorial anaplastic ependymoma	Proton focal	59.4 GyRBE (right frontal lobe)	—	54 GyRBE (right frontal cavity); Cs-131 brachytherapy (resection cavity); 25 GyRBE/5 fx (right temporal cavity)	—	WBRT incl. C1 4 Gy, then CSI 30 Gy/3 Gy
12	Supratentorial anaplastic ependymoma	Photon focal	59.4 Gy (right cingulate gyrus)	—	None		CSI 36 Gy/1.8 Gy; SIB to 54 Gy/2.25 Gy
13	ATRT	Proton focal	50.4 GyRBE (posterior fossa)	—	SRT 30 Gy/5 fx (3 lesions)	—	CSI 25 Gy/2.5 Gy; SIB to 30 Gy/3 Gy
14	Germ cell tumor (pure germinoma at Dx)	Proton CSI	10.8 GyRBE; boost to 21.6 GyRBE	10.8 GyRBE	Proton CSI 18 GyRBE; local boost to 48.6 GyRBE	Proton CSI 18 GyRBE	CSI 21.6 Gy/1.8 Gy; boost to 45 Gy (brain)
**Re-WBRT (n = 9)**							
15	Medulloblastoma	Proton CSI	36 GyRBE; boost to 54 GyRBE	36 GyRBE	None		WBRT 24 Gy/2 Gy; SIB to 30 Gy/2.5 Gy
16	Medulloblastoma, LC/A	Proton CSI	36 GyRBE; boost to 54 GyRBE	36 GyRBE	None		WBRT 24 Gy/2 Gy; SIB to 39 Gy/3.25 Gy
17	Medulloblastoma	Proton CSI	23.4 GyRBE; boost to 54 GyRBE	23.4 GyRBE	None		WBRT 30.6 Gy/1.8 Gy

Pt	Histology	Initial RT			Additional RT between initial RT and re-irradiation		Re-irradiation (dose/dose per fraction)
		Technique	Brain	Spine	Brain	Spine	
18	Medulloblastoma	Photon CSI	36 Gy; boost to 55.8 Gy	36 Gy	SRS 8 Gy (pituitary stalk)	—	WBRT + C4–T4 25 Gy/2.5 Gy
19	HGG (anaplastic oligoastrocytoma)	Proton focal	59.4 GyRBE (left temporal lobe)	—	None		WBRT 25 Gy/2.5 Gy
20	DIPG	Photon CSI	36 Gy; boost to 54 Gy (brainstem)	36 Gy	None		WBRT 24 Gy/2 Gy + focal spine (T10–T12, L4–sacrum)
21	DMG (brainstem)	Photon focal	54 Gy (brainstem)	—	None		WBRT 24 Gy/2 Gy; SIB to 30–36 Gy/2.5–3 Gy
22	Pineoblastoma	Proton CSI	23.4 GyRBE; boost to 54 GyRBE	23.4 GyRBE	SRT 25 GyRBE/5 fx (Meckel's cave)	Proton 30 GyRBE/10 fx (L5–S2)	WBRT 20 Gy/2 Gy
23	CNS embryonal tumor, NOS	Photon CSI	36 Gy	36 Gy	—	20 Gy/5 fx (T12–L5)	WBRT 20 Gy/2 Gy

Abbreviations: ATRT, atypical teratoid/rhabdoid tumor; CSI, craniospinal irradiation; Cs-131, cesium-131; D/N, desmoplastic/nodular; DIPG, diffuse intrinsic pontine glioma; DMG, diffuse midline glioma; Dx, diagnosis; fx, fraction; HGG, high-grade glioma; LC/A, large cell/anaplastic; NOS, not otherwise specified; PF, posterior fossa; RBE, relative biological effectiveness; RT, radiotherapy; SIB, simultaneous integrated boost; SRS, stereotactic radiosurgery; SRT, stereotactic radiotherapy; WBRT, whole brain radiotherapy. Proton doses are in Gy(RBE). Brain includes the brainstem; spine includes the spinal cord and thecal sac. For CSI, the craniospinal dose is the dose to the whole brain and spinal axis, and boost doses are cumulative (total) doses to the boost volume; focal doses are total doses to the tumor or tumor bed (location in parentheses). —, region not included in the treatment field. Additional RT includes any course delivered after completion of the initial course and before re-irradiation with WBRT or CSI.

Table S2. Characteristics, treatment details, and outcomes of the four patients treated with curative-intent re-CSI.

Pt	Age at Dx (y)/ Sex	Histology	Initial RT	Time to 1st relapse (mo)	Additional RT before re-CSI	Surgery at relapse	Disease pattern at re-RT	Systemic therapy before re-RT	Re-CSI (CS dose/fx; boost)	Systemic therapy after re-RT	PFS (mo)	OS (mo)	Status at last follow-up	Notable toxicity
1	19.7/M	Pineoblastoma	Proton CSI 23.4 Gy; boost to 54 Gy	35.6	None	GTR	Oligometastatic	No	CSI 30.6 Gy/1.8 Gy; boost to 54 Gy/2.16 Gy (45 Gy at brainstem)	Yes	17.5*	17.5	Alive, NED	G3 lymphopenia
2	3.7/F	Medulloblastoma, desmoplastic/nodular, PTCH1-mutant	Proton CSI 18 Gy; boost to 54 Gy	92.5	None	STR	Multifocal	No	CSI 30.6 Gy/1.8 Gy; boost to 54 Gy	Yes	106.8*	106.8	Alive, NED	None recorded
3	11.0/F	Supratentorial anaplastic ependymoma	Photon focal 59.4 Gy	12.4	None	None	LMD	No	CSI 36 Gy/1.8 Gy; SIB to 54 Gy/2.25 Gy	Yes	5.8*	5.8	Alive, no progression	G3 symptomatic brain necrosis (steroids, bevacizumab); G3 leukopenia; G4 lymphopenia
4	17.0/M	Germ cell tumor (pure germinoma at diagnosis; AFP elevated at relapse)	Proton CSI 10.8 Gy + local boost 21.6 Gy	15.9	Proton CSI 18 Gy + local boost 30.6 Gy (total 48.6 Gy)	None	LMD	Yes	CSI 21.6 Gy/1.8 Gy; boost to 45 Gy (brain)	No	23.3*	23.3	Alive, NED	G1 radiographic necrosis (asymptomatic); G4 thrombocytopenia/lymphopenia; G3 leukopenia/neutropenia/anemia

Abbreviations: AFP, alpha-fetoprotein; CS, craniospinal; CSI, craniospinal irradiation; Dx, diagnosis; F, female; fx, fraction; G, CTCAE grade; GTR, gross total resection; LMD, diffuse leptomeningeal dissemination; M, male; NED, no evidence of disease; Oligometastatic, solitary metastatic lesion or limited subcentimeter foci of dissemination; OS, overall survival; PFS, progression-free survival; RT, radiotherapy; SIB, simultaneous integrated boost; STR, subtotal resection. Doses are total dose/dose per fraction; initial RT boost doses are cumulative (total) doses. PFS and OS are measured from the start of re-CSI; time to first relapse is measured from completion of initial RT. *No progression at last follow-up. Hematologic toxicity is reported for patients with available laboratory monitoring; "None recorded" indicates no grade ≥3 toxicity documented.
